# High rate of drug resistance among tuberculous meningitis cases in Shaanxi province, China

**DOI:** 10.1038/srep25251

**Published:** 2016-05-04

**Authors:** Ting Wang, Guo-Dong Feng, Yu Pang, Jia-Yun Liu, Yang Zhou, Yi-Ning Yang, Wen Dai, Lin Zhang, Qiao Li, Yu Gao, Ping Chen, Li-Ping Zhan, Ben J Marais, Yan-Lin Zhao, Gang Zhao

**Affiliations:** 1 Department of Neurology, Xijing Hospital, the Fourth Military Medical University, no.169 Changle West Road, Xi’an, Shaanxi, 710032, P.R.China; 2 Department of Neurology, Kunming Medical University affiliated Yan’an Hospital, 245 Renming East Road, Kunming, Yunnan, 650200, P.R.China; 3 National Center for Tuberculosis Control and Prevention, Chinese Center for Disease Control and Prevention, no.155 Changbai Road, Beijing, 102206, P.R.China; 4 Department of Inspection, Xijing Hospital, Fourth Military Medical University, no.169 Changle West Road, Xi’an, Shaanxi, 710032, P.R.China; 5 The Children’s Hospital at Westmead and the Marie Bashir Institute for Infectious Diseases and Biosecurity, University of Sydney, Australia

## Abstract

The clinical and mycobacterial features of tuberculous meningitis (TBM) cases in China are not well described; especially in western provinces with poor tuberculosis control. We prospectively enrolled patients in whom TBM was considered in Shaanxi Province, northwestern China, over a 2-year period (September 2010 to December 2012). Cerebrospinal fluid specimens were cultured for *Mycobacterium tuberculosis*; with phenotypic and genotypic drug susceptibility testing (DST), as well as genotyping of all positive cultures. Among 350 patients included in the study, 27 (7.7%) had culture-confirmed TBM; 84 (24.0%) had probable and 239 (68.3%) had possible TBM. DST was performed on 25/27 (92.3%) culture positive specimens; 12/25 (48.0%) had “any resistance” detected and 3 (12.0%) were multi-drug resistant (MDR). Demographic and clinical features of drug resistant and drug susceptible TBM cases were similar. Beijing was the most common genotype (20/25; 80.0%) with 9/20 (45%) of the Beijing strains exhibiting drug resistance; including all 3 MDR strains. All (4/4) isoniazid resistant strains had mutations in the *katG* gene; 75% (3/4) of strains with phenotypic rifampicin resistance had mutations in the *rpoB* gene detected by Xpert MTB/RIF®. High rates of drug resistance were found among culture-confirmed TBM cases; most were Beijing strains.

Tuberculous meningitis (TBM) is the most severe form of extrapulmonary tuberculosis (TB)^1^. Diagnosis is difficult because clinical features are non-specific and laboratory tests are insensitive^2^. Culture remains the reference standard, but traditional solid media (Lowenstein-Jensen; L-J) culture methods are slow (4–8 weeks) and have poor sensitivity. In 2007 the World Health Organization (WHO) recommended that Mycobacteria Growth Indicator Tube (MGIT) 960 liquid culture techniques should replace conventional solid culture methods^3^, offering a more rapid and reliable tool for the diagnosis of TB and for drug susceptibility testing (DST). However, in China, the MGIT960 culture system has not been established in routine clinical practice and few hospitals have the ability to culture *M. tuberculosis* from cerebrospinal fluid (CSF).

Drug-resistant TB is an emerging problem with epidemic spread of drug-resistant strains recorded in multiple settings^4^. WHO estimates that 9.6 million people developed tuberculosis in 2014, of whom 480,000 (5.0%) had multidrug resistant (MDR; resistance against isoniazid and rifampicin) disease^5^. The majority of MDR- tuberculosis cases are located in the Indian subcontinent, the Russian Federation and China, with high caseloads also reported from Southern Africa where many patients are co-infected with human immunodeficiency virus (HIV)^5^. In China, a 2007 national drug-resistance survey showed that among new TB cases, 34.2% were resistant to any first-line tuberculosis drug; among 3037 new and 892 previously treated cases, 5.7% and 25.6%, respectively had MDR tuberculosis^6^. However, no drug resistance survey has focused specifically on the western parts of China where access to antituberculosis drugs are poorly regulated and tuberculosis disease rates are highest.

Phenotypic DST assesses the ability of isolated bacilli to grow in the presence of a “critical concentration” of the test drug^7^. The critical concentration is defined as the lowest drug concentration that inhibits ≥95% of wild-type *M. tuberculosis* strains^7^, but testing is time consuming to perform. The detection of specific mutations associated with drug resistance (genotypic DST) may overcome some of the limitations, especially the long time delays, associated with phenotypic DST^8^. Drug resistance related mutations have been found in the *katG, inhA, rpoB, embB, gyrA, gyrB, rrs-KAN, eis, rpsL* and *gidB* genes^9^. The novel Xpert MTB/RIF® assay has the ability to simultaneously detect *M. tuberculosis* and *rpoB* gene mutations in a critical 81 base pair region (amino acid 507–533), referred to as the rifampicin-resistance-determining region (RRDR)^10^. In general, the presence of rifampin resistance is highly suggestive of MDR tuberculosis, although rifampin mono-resistance is well documented and fairly common in some settings^11^.

Molecular epidemiological analysis helps to characterize the *M. tuberculosis* population structure and transmission dynamics in a particular setting. Mycobacterial interspersed repetitive unit (MIRU) typing, is currently the most reproducible and widely used method. MIRU typing assesses variable number tandem repeats found in 41 loci across the *M. tuberculosis* genome; its discriminatory power varies depending on the number and combination of loci used^12^. Spacer oligonucleotide typing (spoligotyping) analyzes genomic polymorphisms in the short direct repeat (DR) region of *M. tuberculosis*, consisting of identical 36-bp DRs interspersed with 35–41 base pair non-repetitive spacer sequences^13^. A combination of spoligo- and MIRU typing is often used for enhanced strain resolution.

The molecular epidemiology and drug resistance profile of *M. tuberculosis* strains isolated from TBM patients have not been documented in northwestern China. Neither has a comparison of the disease phenotype observed in drug resistant and drug susceptible cases, or a detailed description of the mutations associated with particular drug resistance patterns, been performed.

## Methods

### Study participants and setting

We performed a prospective descriptive study over a 2-year study period (September 2010 to December 2012) in Shaanxi province, China. Patients with meningitis (fever, headache and nuchal rigidity or abnormal CSF parameters) were recruited from all tertiary referral centres in Shaanxi province (12 hospitals in total), China; coordinated by the Fourth Military Medical University and Xijing hospital. Shaanxi province is the economic and political centre of the northwestern part of China with a population of 36.7 million people. The TB incidence is high (notification rate 109/100,000 population)^14^,^15^ and HIV-1 prevalence is low (<1% of TB patients)^16^. Two trained interviewers collected clinical data with the use of a standard questionnaire. Additional clinical information was gathered from reviewing relevant clinical notes. Patients were scored according to the revised British Medical Research Council TBM severity grade^17^ and classified according to uniform TBM diagnostic criteria^18^, as ‘definite’, ‘probable’ or ‘possible’ TBM. All patients were tested for HIV-specific antibodies (Roche Elecsys HIV Combi).

### Specimen processing

All CSF specimens were sent to a central laboratory at Xijing hospital, where study tests were performed. Conventional and modified Ziehl-Neelsen stain^19^, standard CSF biochemical and microbiological tests, including Gram stain, India ink stain and ESAT-6 (early secreting antigen target 6KD) immunocytochemical stain, bacterial culture and liquid medium culture, were performed on all specimens. MGIT 960 culture (Becton, Dickinson and Co., Franklin Lakes, NJ) was performed using 0.5 ml of CSF. Culture positive isolates were sent to the National Tuberculosis Reference Laboratory (NTRL) at the Chinese Center for Disease Control and Prevention, Beijing, for *M. tuberculosis* confirmation by *16S rRNA* gene sequencing^20^ (Table S1), drug susceptibility testing (DST) and genotyping.

### Drug susceptibility testing and genotyping

Phenotypic DST was performed according to standard guidelines using the L-J proportional method^21^, for isoniazid (H), rifampin (R), ethambutol (E), streptomycin (S), kanamycin (K), amikacin (A), capreomycin (C), levofloxacin (Lfx), para-aminosalicylic acid (PAS) and prothionamide (Pto). Testing of H, R, E, S, K, A, C, moxifloxacin (Mfx), Lfx, PAS, Pto were also done using MGIT 960^22^ to determine the mean inhibitory concentration (MIC)^23^. Minimum inhibitory concentration (MIC) testing quantifies the degree of drug resistance detected, by determining the drug concentration at which >99% of organism growth is suppressed^24^. Critical concentrations and MIC determination techniques are poorly validated for most second-line TB drugs^25^. Genotypic DST was performed using standard primers for the *inhA, katG, rpoB, embB, gyrA, gyrB, rrs-KAN, eis, rpsL, gidB* genes^26^,^27^,^28^,^29^ (Table S2); sequences were compared to the *M. tuberculosis* reference strain H37Rv and deposited in GenBank (http://www.ncbi.nlm.nih.gov/BLAST/). In addition, gene Xpert MTB/RIF® was performed according to standard protocol^10^.

DNA was extracted from freshly cultured *M. tuberculosis* colonies on solid (L-J) medium and processed using standard reagents (Tiangen Biotech ltd., Beijing) and methodology for spoligo^13^ and 25-loci MIRU-25 typing^12^. Given that the Hunter and Gaston Discriminatory Index (HGDI) of some loci included in standard 24-loci MIRU is low^30^, we chose 25 loci with maximal (HGDI = 99.7%) discriminatory power (Table S3).

### Patient management

TBM patients were given 3 months of daily oral isoniazid (300–400 mg/d), rifampin (450–600 mg/d), pyrazinamide (25–40 mg/kg/d) and ethambutol (15–20 mg/kg/d) or intramuscular streptomycin (15 mg/kg/d) during the intensive phase of treatment. The same daily dose of isoniazid and rifampin were given for a further 6 months during the continuation phase of treatment. Additional treatment with levofloxacin 1000 mg/d or moxifloxacin 800 mg/d was included at the discretion of the treating physician, as were adjunctive treatment with other antibiotics (meropenem, cefoperazone and/or vancomycin) or supportive therapy (acetazolamide, corticosteroids, mannitol, or CSF shunting).

### Data analysis and ethics

Statistical analyses were performed with SPSS (V21.0, IBM) and GraphPad Prism v.5.0 (GraphPad Software Inc., La Jolla, CA). Continuous variables were described with medians and ranges, categorical variables with numbers and percentages. Univariate analysis was performed using the Mann-Whitney U test for nonparametric data and between group differences assessed using the Chi square test. Differences were considered significant with p-values <0.05. MIRU-25 and spoligotyping clustering patterns were analyzed with BioNumerics software version 5.0 (Applied Maths, Sint-Martens-Latem, Belgium). The discriminatory power of various MIRU loci was calculated using the HGDI^31^. *M. tuberculosis* H37Rv (ATCC 25177, obtained from US-CDC) was used as the reference strain and for quality control purposes.

### Ethics Statement

The study protocol was approved by the Ethics Committee of Xijing Hospital (Fourth Military Medical University) and written informed consent was obtained from all participants or their legal surrogates. The methods were carried out in accordance with the approved guidelines.

## Results

**Figure 1:**
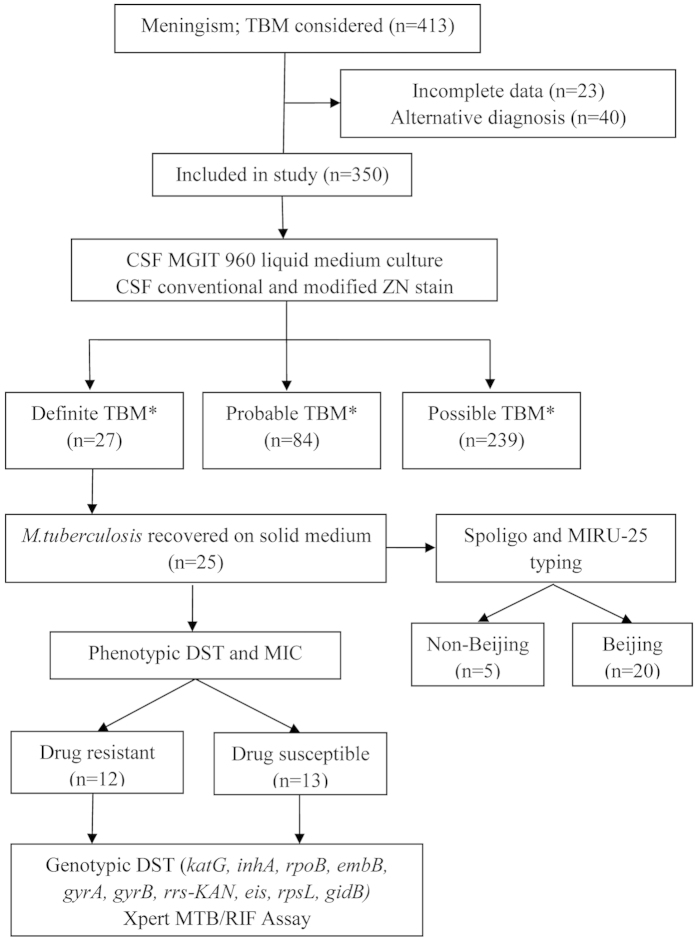
Flow diagram of patients in whom tuberculous meningitis was considered and special investigations done. TBM-tuberculous meningitis; *M. tuberculosis*-*Mycobacterium tuberculosis*; ZN-Ziehl-Neelsen; CSF-Cerebral Spinal Fluid; DST-Drug Susceptibility Testing; MIC-Minimal Inhibitory Concentration; Spoligo- spacer oligonucleotide; MIRU-25-25 locus Multiple Interspersed Repetitive Unit; *Classification according to consensus uniform research case definition criteria^18^.

In total 350 patients were included in the analysis (Fig. 1); 27 (7.7%) with definite TBM, 84 (24.0%) with probable and 239 (68.3%) with possible TBM. None of the patients were HIV-infected. The majority were male (205/350; 58.6%) with a median age of 32 years (range 1–87 years). Table 1 provides an overview of relevant clinical, CSF, central nervous system (CNS) imaging and extra-neural findings. Two culture-confirmed TBM patients had normal CSF protein and sugar values, with leukocyte counts of 7/μl and 233/μl (lymphocytes 45% and 85%) respectively. Both had chest radiographs suggestive of pulmonary TB. In total, only 93 (26.6%) patients had a chest radiograph findings suggestive of TB.

Of 27 positive CSF *M. tuberculosis* cultures, 25 (92.6%) were recovered on L-J solid media and subjected to DST. The majority of these patients 19 (76%) had chest radiograph findings suggestive of TB; median age 26 years (range 3–82 years); 68.0% (17/28) were female. Among positive isolates nearly half (12/25; 48.0%) demonstrated phenotypic drug resistance. The demographic profile and clinical features of TBM patients with and without phenotypic drug resistance are summarized in Table 2. Hospital outcomes were comparable; 2/12 (16.7%) cases with drug resistant disease (mono-S, mono-K) died in hospital and 3/13 (23.1%) of those with drug susceptible disease; 3/12 cases with drug resistant disease had moxifloxacin added to the treatment regimen. All 3 MDR cases had a good outcome. Case #18 received moxifloxacin and meropenem in addition to the standard TB treatment, while cases #11 and #19 received standard TB treatment with cefoperazone or meropenem and vancomycin; no second-line injectables were used.

**Figure 2:**
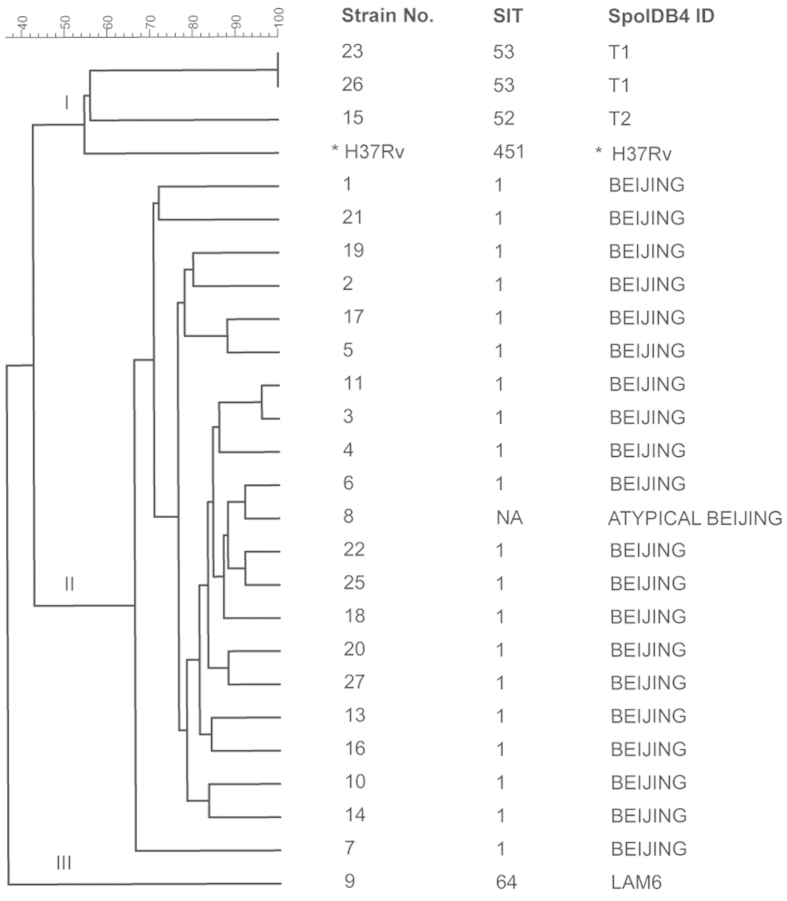
MIRU-25 phylogenetic relationship of *M. tuberculosis* genotypes identified in TBM patients; with associated spoligotype. MIRU-25–25 locus Multiple Interspersed Repetitive Unit typing as defined in Methods; Strain No.- Liquid MGIT960 culture positive TBM isolates were assigned numbers 1–27 (2 isolates were not recovered successfully on Lowenstein-Jensen solid medium); SIT - Spoligo International Type; SpoIDB4.0-Fourth International Spoligotyping Database; SpolDB4 ID - Identification from the SpoIDB4.0 database; NA-Unclassified in SpoIDB4.0 database, we defined it as an atypical Beijing strain; *Reference strain.

Drug resistance patterns observed in TBM patients infected with different *M. tuberculosis* genotypes are reflected in Table 3. Beijing was the dominant genotype (20/25; 80.0%) identified and was associated with high rates of drug resistance (45%; 9/20). This included all 3 MDR strains; 1 with pre-XDR (MDR with additional resistance to either fluoroquinolones or second-line injectable agents). Figure 2 provides more genotypic detail and assesses the phylogenetic relationship of the various *M. tuberculosis* genotypes identified. There was no evidence of genotype clustering suggestive of common source exposure or laboratory contamination.

Table 4 summarizes all the mutations detected in genes associated with drug resistance with corresponding MIC values. All strains with phenotypic isoniazid resistance had mutations in *katG* (S315T, R463L) and high MIC values (>32.0 μg/ml). Most (3/4) strains with phenotypic rifampin resistance had mutations in *rpoB*, all of these mutations occurred in 81-base pair RRDR which could be detected by Gene Xpert MTB/RIF ®. A single case with phenotypic low-level mono-rifampin resistance was not detected by Gene Xpert MTB/RIF ®; no mutation could be identified on full sequencing of the *rpoB* gene. Pan-resistance to all injectable drugs was observed in strains with mutations in *rpsL*, *gidB* and *rrs-KAN*. No phenotypic fluoroquinolone resistance was detected, despite the presence of non-synonymous mutations in the *gyrA* gene.

## Discussion

Given the absence of representative population-based data and the limited number of bacteriologically confirmed TBM cases, it is difficult to comment on the TBM incidence within the study population. However, the fact that nearly half of the TBM patients with positive CSF cultures were infected with a resistant *M. tuberculosis* strain is alarming. A study from Xijing Hospital in Shaanxi province evaluated a collection of 90 strains, isolated during 2009–2012 from pulmonary tuberculosis cases, and identified MDR in 46.7% (42/90) of isolates; 20% (18/90) were isoniazid mono- and 2.2% (2/90) were rifampin mono-resistant^32^. These isolates were recovered from a select group of referred patients and were not necessarily representative of strains transmitted within the community. However, with high rates of drug resistant TB among pulmonary TB cases it is expected that similar rates will be observed among TBM cases if these strains are readily transmitted. In fact, the high rates of drug resistance found among *M. tuberculosis* strains isolated from TBM patients referred from all over Shaanxi province, suggest that these strains are widely dispersed and readily transmitted within the wider community.

There is now convincing evidence of epidemic spread of drug-resistant *M. tuberculosis* strains in multiple settings. A study of new TBM cases in south western China^33^ reported an even higher rate (18%) of MDR-TBM than what we detected. As in previous studies^34^,^35^, we found the clinical features of drug-resistant and -susceptible TBM to be similar, which emphasizes the importance of routine DST or to identify drug-resistant TBM cases. The current study did not identify any TBM cases in children <3 years of age, which is the age group at highest risk of TBM following *M. tuberculosis* infection^36^. However, most children would have been sent to specialized pediatric hospitals that did not contribute to study recruitment. In general, pediatric TB case rates are relatively low in Shaanxi province, which may be a consequence of good BCG coverage, unique population demographics with limited exposure of young children or missed diagnoses. It is estimated that 76% of infants are BCG vaccinated at birth in China^37^. Data from Shaan’xi Province are limited, but a review of BCG vaccination and scar rates conducted from 1992 to 2001 suggested excellent BCG coverage (97%)^38^.

The good outcomes achieved in our TBM cohort may be related to early treatment initiation, non-severe disease at presentation, excellent treatment adherence and the absence of HIV co-infection. The fact that all 3 MDR-TBM patients improved is unexpected, given that MDR-TBM is usually associated with a poor prognosis^39^. All 3 cases were immunocompetent, were diagnosed early, and received additional treatment with antibiotics that has known activity against *M. tuberculosis*^40^. Typically the CSF in TBM reflects a pleocytosis with raised protein (>1mg/dl) and reduced sugar^17^. However, a number of patients did not meet these criteria, including two cases with culture-confirmed TBM. Given the ancillary evidence of TBM, these specimens are unlikely to have been contaminated. Similar observations have been made in India^41^, where only 80% of TBM patients had the classic CSF triad of reduced sugar, raised protein (>1mg/dl) and pleocytosis (total leucocyte count of >20 cells/ml). This emphasizes the need to consider TBM even in patients with initial mild clinical symptoms and minor CSF abnormalities. The fact that a high percentage of patients with culture-confirmed drug resistant TBM was female and had chest radiograph abnormalities has not been observed before and is difficult to explain mechanistically.

Mono-drug resistance against streptomycin and other injectable drugs has also been found in other settings^42^. It may be explained by discrepant adherence to oral and injectable drugs during TB retreatment, or prolonged courses of an injectable agent given for other indications. MDR strains that includes streptomycin resistance are common among patients failing first-line treatment in Mongolia, indicating likely transmission of these strains given the absence of previous streptomycin exposure^43^. Mono-resistance against para-aminosalicylic acid and prothionamide is difficult to explain, given the poor potency of these drugs and guidance to only use them as part of an MDR-TB regimen, but have been reported in previous Asian studies^44^,^45^. Inappropriate antibiotic use is a major challenge in China^46^ and all of Asia and since access to second-line TB drugs is poorly regulated exposure to these drugs may have occurred outside well supervised programs.

The 25-loci MIRU used in our study showed good discriminatory power, even among Beijing strains in which strain resolution is poor with standard 24-loci MIRU typing^47^. The strain population structure was similar to those observed among pulmonary TB patients in China^48^ without any genotype clustering suggestive of common source exposure or laboratory contamination. The position of an atypical Beijing strain in the middle of the Beijing phylogenetic tree is unexpected, suggesting that MIRU-25 strain resolution remains sub-optimal despite HGDI optimization. There is a possibility that DRs may be subject to cycling convergent evolution that confuse the strain specific signal; especially within highly monomorphic Beiing lineage strains^49^. TBM caused by Beijing genotype strains has been associated with a higher risk of drug resistance^50^, similar to findings in patients with pulmonary TB^51^. A review of osteo-articular TB cases diagnosed in Beijing, China, identified any drug resistance in more than half of the isolates (62/113; 54.9%) ; MDR was present in 15.0% (17/113). The vast majority of strains were of the Beijing lineage; 80.5% SIT 1^52^.

Rifampin mono-resistance has been reported with at relatively high frequency among new cases in Turkey^53^; rising rates of rifampin mono-resistance has also been reported in parts of South Africa^54^. We detected a single case of rifampin mono-resistance that tested negative on Gene-Xpert MTB/RIF®, but acknowledge that phenotypic DST can be inaccurate close to the cutoff levels defining resistance^8^,^55^. All three MDR strains in our study tested positive for rifampin resistance by Gene-Xpert MTB/RIF®. The fact that *rpsL* (K43R) was associated with high-level streptomycin resistance, but was also detected in 8% (2/24) of streptomycin-sensitive strains, opens the possibility of multiple gene interactions defining a resistant phenotype and demonstrates the hazards of deducing drug resistance phenotypes from simplistic mutation analyses^56^,^57^. The only other mutation found in the *rpsL* gene was a silent mutation in codon 39 (T39T), which has not been associated with streptomycin resistance^58^. Similarly, *gidB* gene mutations (E92D and A205A) were not associated with drug resistance in our study, but *gidB* L16R mutations have been associated with resistance in Latin American-Mediterranean lineage strains^58^. The *KatG* (S315T) mutation is the most common mutation causing high-level isoniazid resistance, while the *KatG* (R463L) variation is thought to be a polymorphism^59^. Numerous *gyrA* mutations have been associated with fluroquinolone resistance, but the S95T *gyrA* mutation that we observed was present in all the TBM strains collected and not associated with drug resistance^26^.

A major study limitation was the small number of culture-confirmed TBM cases; unfortunately this is the reality in all TBM studies^60^,^61^, especially those enrolling HIV-uninfected TBM patients^33^,^62^,^63^. Culture yield may have been further compromised by delays in processing time and the common use of empiric antibiotics before a CSF sample is collected^64^. However, the study cohort represents one of the largest HIV-uninfected TBM cohorts to date. Our study was also limited by its geographic restriction, but findings should be relevant to greater western China and the surrounding region. Clinical, drug resistance and genotypic data from TBM patients in different parts of China would be highly informative. This may be achieved by a nationally coordinated TBM surveillance project to follow-up on our findings.

In conclusion, high rates of drug resistance were found among culture-confirmed TBM cases in Shaanxi Province, China. Findings suggest primary transmission of drug-resistant Beijing strains within the community, which will require accurate identification and treatment of drug resistant pulmonary TB cases to limit transmission. Routine DST is also indicated to optimize the treatment of TBM cases, since clinical features do not differentiate drug resistant and drug susceptible cases.

## Additional Information

**How to cite this article**: Wang, T. *et al.* High rate of drug resistance among tuberculous meningitis cases in Shaanxi province, China. *Sci. Rep.* **6**, 25251; doi: 10.1038/srep25251 (2016).

## Supplementary Material

Supplementary Information

## Figures and Tables

**Table 1:** Overview of uniform research case definition criteria^18^ in confirmed, probable and possible TBM cases.

Diagnostic categories and criteria (Maximum category score)	Diagnostic score	Confirmed n = 25 (%)	Probable n = 84 (%)	Possible n = 239(%)
Clinical (Maximum score = 6)				
CNS symptom duration >5 days	4	25 (100.0)	67 (79.8)	159 (66.5)
Other symptoms suggestive of TB^a^	2	19 (76.0)	43 (51.2)	31 (13.0)
Recent close contact with an infectious TB case^b^ or positive TST/IGRA	2	11 (44.0)	9 (10.7)	5 (2.1)
Focal neurological deficit^c^	1	14 (56.0)	34 (40.5)	95 (39.7)
Cranial nerve palsy	1	4 (16.0)	5 (6.0)	24 (10.0)
Altered consciousness	1	18 (72.0)	45 (53.6)	108 (45.2)
CSF (Maximum score = 4)				
Clear appearance	1	18 (72.0)	77 (91.7)	221(92.5)
Cells: 10–500 per μl	1	24 (96.0)	72 (85.7)	210 (87.9)
Lymphocytic predominance (>50%)	1	14 (56.0)	61 (72.6)	192 (80.3)
Protein concentration greater than 1 g/L	1	17 (68.0)	54 (64.3)	72 (30.1)
Glucose concentration <2.2 mmol/L	1	22 (88.0)	52 (61.9)	53 (22.2)
CNS imaging (Maximum score = 6)				
Hydrocephalus	1	11 (44.0)	22 (26.2)	27 (11.3)
Basal meningeal enhancement	2	9 (36.0)	18 (21.4)	23 (9.6)
Tuberculoma	2	3 (12.0)	1 (1.2)	0
Infarct	1	6 (24.0)	6 (7.1)	11 (4.6)
Pre-contrast basal hyperdensity	2	4 (16.0)	12 (14.3)	8 (3.3)
TB elsewhere (Maximum score = 4)				
CXR suggestive (PTB = 2; miliary TB = 4)	2/4	19 (76.0)	43 (51.2)	31(13.0)
Other imaging suggestive of TB	2	11 (44.0)	9 (10.7)	5 (2.1)
Positive AFB, *M. tuberculosis* or NAAT from extra-neural specimen	4	4 (16.0)	5 (6.0)	2 (0.8)
Score; median (range)	20 max	13 (6–20)	12 (10–18)^d^	6 (6–11)^e^

TBM-tuberculous meningitis; TB-tuberculosis; PTB-pulmonary TB: TST-Tuberculin Skin Test; IGRA-Interferon Gamma Release Assay; CSF-Cerebral Spinal Fluid; CNS-Central Nervous System; CXR-Chest X-ray; AFB-Acid Fast Bacilli; NAAT-commercial Nucleic Acid Amplification Test.

^a^Weight loss (or poor weight gain in children), night sweats, or persistent cough for more than 2 weeks.

^b^History of recent (within past year) close contact with an individual with pulmonary TB.

^c^Excluding cranial nerve palsies.

^d^Scores of 10 and 11 when brain imaging was not available.

^e^Scores of 10 and 11 when brain imaging was available.

**Table 2:** Demographic profile and clinical features of culture-confirmed TBM patients with and without phenotypic drug resistance.

Variable	Drug resistant^a^ = 12 (%)	Drug susceptibleN = 13 (%)	TotalN = 25 (%)
Median age; years (range)	26 (3–65)	27 (3–82)	26 (3–82)
Female	8 (66.7)	9 (69.2)	17 (68.0)
History			
Fever	9 (75.0)	10 (76.9)	19 (76.0)
Headache	9 (75.0)	11 (84.6)	20 (80.0)
Vomiting	2 (16.7)	5 (38.5)	7 (28.0)
Seizures	2 (16.7)	2 (15.4)	4 (16.0)
Previous TB treatment	4 (33.3)	4 (30.8)	8 (32.0)
MRC grading^b^			
Grade 1	2 (16.7)	2 (15.4)	4 (16.0)
Grade 2a	2 (16.7)	1 (7.7)	3 (12.0)
Grade 2b	6 (50.0)	6 (46.2)	12 (48.0)
Grade 3	2 (16.7)	4 (30.8)	6 (24.0)
Imaging			
Chest X-ray suggestive of TB	10 (83.3)	9 (69.2)	19 (76.0)
Hydrocephalus	5 (41.7)	6 (46.2)	11 (44.0)
Basal meningeal enhancement	4 (33.3)	5 (38.5)	9 (36.0)
Infarcts	2 (16.7)	4 (30.8)	6 (24.0)
CSF findings			
Total leukocyte count cells/μl; median (range)	225 (27–683)	101 (7–319)	143 (7–683)
Lymphocytes >50%	6 (50.0)	8 (61.5)	14 (56.0)
Protein mg/dl; median(range)	1.3 (0.2–3.9)	1.6 (0.9–3.8)	1.4 (0.2–3.9)
Protein >1.0 mg/dl	7 (58.3)	10 (76.9)	17 (68.0)
Glucose mmol/l; median(range)	1.5 (0.4–3.6)	1.6 (0.6–3.8)	1.5 (0.4–3.8)
Glucose <2.2 mmol/l	10 (83.3)	12 (92.3)	22 (88.0)
Death before hospital discharge	2 (16.7)	3 (23.1)	5 (20.0)

TBM-tuberculous meningitis; TB-tuberculosis.

^a^Drug resistant-phenotypic resistance against any TB drug tested (isoniazid, rifampin, ethambutol, streptomycin, kanamycin, amikacin, capreomycin, levofloxacin, moxifloxacin, p-aminosalicylic acid, prothionamide).

^b^British Medical Research council disease severity grade^17^; Grade 1, Glasgow Coma Scale (GCS) of 15 without focal neurologic signs; Grade 2, GCS 11–14 with a) no focal neurological signs and b) with focal neurological signs; Grade 3, GCS ≤10 with or without focal neurological deficit.

**Table 3:** Drug resistance patterns observed in TBM patients infected with different *M. tuberculosis* genotypes.

Spoligotype SpoIDB4.0^a^ (N)	Spoligotype fingerprint	Drug resistance profile			
		Mono^b^ (n)	MDR^c^ (n)	XDR^d^ (n)	Other^e^ (n)
BEIJING (20)	□□□□□□□□□□□□□□□□□□□□□□□□□□□□□□□□□□■■■■■■■■■	Mono-H:1	3	0	0
		Mono-E:1			
		Mono-S:2			
		Mono-Pto:1			
		Mono-PAS:1			
ATYPICAL BEIJING (1)	□□□□□□□□□□□□□□□□□□□□□□□□□□□□□□□□□□■■■■□□□■■	0	0	0	–
T1 (2)	■■■■■■■■■■■■■■■■■■■■■■■■■■■■■■■■□□□□■■■■■■■	0	0	0	C, A, K
T2 (1)	■■■■■■■■■■■■■■■■■■■■■■■■■■■■■■■■□□□□■■■□■■■	Mono-R:1	0	0	–
LAM6 (1)	■■■■■■■■■■■■■■■■■■■■□□□□■■■■□■■■□□□□■■■■■■■	Mono-Km:1	0	0	–

^a^SpoIDB4.0, Fourth International Spoligotyping Database.

^b^Mono drug resistant, H-isoniazid, E-ethambutol, Pto- prothionamide, S- streptomycin, PAS- para-aminosalicylic acid, R- rifampicin, K-kanamycin; C- capreomycin; A- amikacin.

^c^MDR-multidrug resistant, resistant to rifampicin and isoniazid.

^d^XDR-extremely drug resistant, MDR with resistance to both fluoroquinolones and second-line injectable agents.

^e^Other- resistant to any other drug combination.

**Table 4:** Drug-resistance mutations and corresponding phenotypic drug resistance detected.

Individual drug	Phenotypic resistance (Strain No.)	Resistance-associated mutations			MIC^a^(Critical concentration^b^) μg/ml
		Genes	Nucleotide change*	Amino acid change	
Isoniazid (4)	H,R,S (#11)	*kat G*	315,AGC→ACC	S→T	>32.0 (4.0)
			463,CGG→CTG	R→L^c^	
	H,R,E (#18)		315,AGC→ACC	S→T	>32.0 (4.0)
			463,CGG→CTG	R→L	
	H,R,C,A,K (#19)		315,AGC→ACC	S→T	>32.0 (4.0)
			463,CGG→CTG	R→L	
	H (#27)		315,AGC→ACC	S→T	>32.0 (4.0)
			463,CGG→CTG	R→L	
	–	*inhA* or promotor	–	–	–
Rifampin (4)	H,R,S (#11)	*rpoB*	430,CTG→CCG^e^	L→P	2.0 (1.0)
	H,R,E (#18)		450,TCG→TTG^e^	S→L	>8.0 (1.0)
	H,R,C,A,K (#19)		452,CTG→CCG^e^	L→P	8.0 (1.0)
	R (#15)^d^		–	–	2.0 (1.0)
Ethambutol (2)	H,R,E (#18)	*embB*	306,ATG→ATA	M→I	20.0 (5.0)
	E (#2)^d^		–	–	>20.0 (5.0)
Quinolones (0)	H,R,E (#18)	*gyrA*	95,AGC→ACC^f^	S→T	Mfx-0.3 (2.0)
			90,GCG→GTG	A→V	Lfx-0.6 (2.0)
	–	*gyrB*	–	–	–
Injectables (6)	H,R,S (#11)	*rpsL*	43,AAG→AGG	K→R	S > 4.0 (2.0)
		*gidB*	92,GAA→GAC^c^	E→D	K-4.0 (5.0)
			205,GCA→GCG^c^	A→A	A-2.0 (4.0)
					C-2.5 (5.0)
	S (#14)	*rpsL*	43,AAG→AGG	K→R	S > 4.0 (2.0)
		*gidB*	92,GAA→GAC	E→D	K-4.0 (5.0)
			205,GCA→GCG	A→A	A-2.0 (4.0)
					C-5.0 (5.0)
	S (#1)	*gidB*	92,GAA→GAC	E→D	S > 4.0 (2.0)
			205,GCA→GCG	A→A	K-8.0 (5.0)
					A-4.0 (4.0)
					C-5.0 (5.0)
	K (#9)	*gidB*	16,CTT→CGT	L→R	S-2.0 (2.0)
					K-16.0 (5.0)
					A-8.0 (4.0)
					C-5.0 (5.0)
	H,R,S,C,A,K (#19)	*gidB*	92,GAA→GAC	E→D	S > 4.0 (2.0)
			205,GCA→GCG	A→A	K > 128.0 (5.0)
		*rrs-KAN*	1401, A→G	–	A > 32.0 (4.0)
					C-20.0 (5.0)
	C,A,K (#26)	–	–	–	S-4.0 (2.0)
					K-32.0(5.0)
					A-8.0 (4.0)
					C-20.0 (5.0)
	−(#16)	*rpsL*	39, ACC→ACT	T→T	S-2.0 (2.0)
		*gidB*	92,GAA→GAC	E→D	K-8.0(5.0)
			205,GCA→GCG	A→A	A-1.0 (4.0)
					C-5.0 (5.0)
	H (#27)	*rpsL*	43, AAG→AGG	K→R	S-2.0 (2.0)
		*gidB*	92,GAA→GAC	E→D	K-4.0(5.0)
			205,GCA→GCG	A→A	A-2.0 (4.0)
					C-5.0 (5.0)
	–	*eis*	–	–	–

MIC-Minimum Inhibitory Concentration; H- isoniazid; R- rifampin; E- ethambutol; Mfx-moxifloxacin; Lfx-levofloxacin; S-streptomycin; K- kanamycin, A-amikacin; C-capreomycin; RRDR-Rifampin Resistance Determining Region.

^a^MIC-Mean Inhibitory Concentration.

^b^Critical concentration as recommended by WHO and CLSI^23^.

^c^Detected in all 20 Beijing and 1 atypical Beijing strain.

^d^Resistance identified by L-J solid DST and MGIT 960 MIC.

^e^L511P, S531L, L533P with *Escherichia coli* numbering (all within the 81 base pair RRDR).

^f^Detected in all the isolates, both drug resistant and drug susceptible; *compared to H37Rv.
