# Supplementary material for: High rate of drug resistance among tuberculous meningitis cases in Shaanxi province, China
Source: Sci Rep. 2016 May 4;6:25251. doi: 10.1038/srep25251 (PMC4855176; doi:10.1038/srep25251)
Supplement: Supplementary Information [file srep25251-s1.doc]

**High rate of drug resistance among tuberculous meningitis cases in Shaanxi province, China**

Ting Wang^1,4¶^ (MD,PhD); Guo-Dong Feng^1¶^ (MD,PhD); Yu Pang^2¶^ (PhD); Jia-Yun Liu^3^ (PhD); Yang Zhou^2^ (PhD); Yi-Ning Yang^1^ (BS); Wen Dai^1^ (BS); Lin Zhang^1^ (MD,PhD); Qiao-Li^1^ (MD,PhD); Yu-Gao^1^(MD,PhD); Ping Chen^1^(MD,PhD); Li-Ping Zhan^4^ (MD,PhD); Ben J Marais^5^ (MD,PhD); Yan-Lin Zhao^2^ (MD,PhD)**^†^**; Gang Zhao^1^**^†^** (MD,PhD)

**^¶^** Joint first authors; these authors contributed equally to this work

^1^Department of Neurology, Xijing Hospital, Fourth Military Medical University; ^2^National Center for Tuberculosis Control and Prevention, Chinese Center for Disease Control and Prevention; ^3^Department of Inspection, Xijing Hospital, Fourth Military Medical University; ^4^Department of Neurology, Kunming Medical University affiliated Yan’an Hospital; ^5^Marie Bashir Institute for Infectious Diseases and Biosecurity, University of Sydney, Australia

**†Address for correspondence**

Dr. Gang Zhao, Department of Neurology, Xijing Hospital, the Fourth Military Medical University, no.169 Changle West Road, Xi’an, Shaanxi, 710032, P.R.China. Email: zhaogang@fmmu.edu.cn.

Or

Dr. Yan-lin Zhao, National Center for Tuberculosis Control and Prevention, Chinese Center for Disease Control and Prevention, no.155 Changbai Road, Beijing, 102206, P.R.China. Email: zhaoyanlin@chinatb.org.

**Running title:** TBM in Shaanxi Province, China

**Key words:** tuberculous; meningitis; genotype; drug resistance; mutation

**Word count:** Abstract 200; Text: 3135

**Conflicts of interest:** None to declare

^#^ This study was supported by National Key Project (2013ZX10003003) and the National Natural Science Foundation of China (81371334).

**TableS1. PCR primer sequences of *16S rRNA* gene and *16S-23S rRNA* internal transcribed spacer (ITS) genes used in *mycobacterium* species identification.**

| **Locus** | **PCR primer pairs^a^** | **Product size** | **Tm (℃)** |
| --- | --- | --- | --- |
| ***16-23467*** | F 5’AGCCAGTGGCCTAACCCTCGG | 450bp | 61 |
|  | R 5’CCGAGGCATATCGCAGCCTCC |  |  |
| ***rrs1690*** | F 5’GGGGCGTGGCCGTTTGTTTT | 1800bp | 61 |
|  | R 5’CACCCGGCTCTCGCCCACTA |  |  |
| ***16s555*** | F 5’GGCGTGCTTAACACATGCAA | 500bp | 60 |
|  | R 5’TCACGAACAACGCGACAAAC |  |  |

PCR-polymerase chain reaction; Tm-annealing temperature

^a^F, forward primer; R, reverse primer.

**Table S2. PCR primer sequences used in drug resistant gene sequencing**

| **Drug** | **Locus** | **PCR primer pairs^a^** | **Product size** | **Tm(℃）** |
| --- | --- | --- | --- | --- |
| **Isoniazid** | ***inhA*** | F 5'-AgAAggTCAACgCCAAC | 934bp | 60 |
|  |  | R 5'-TgTgTgCAgCTCgAgTAACC |  |  |
|  | ***katG*** | F 5'-AATCGCGCCGGGCAAA | 1000bp | 60 |
|  |  | R 5'-GGTCCCTGCGGTCAGC |  |  |
|  |  | F 5’-TGAGACAGTCAATCCCGATGC | 1015bp | 60 |
|  |  | R 5’-TAACAGCTGGCCCGACAAC |  |  |
|  |  | F 5’-GTTGCCGGCGAAAACAATCA | 701bp | 60 |
|  |  | R 5’-CGGGGTTATCGCCGATGT |  |  |
| **Rifampin** | ***rpoB*** | F 5'-GCCGGCCGAAACCGA | 1000bp | 60 |
|  |  | R 5’-CGTAGCGCTTCTCCTTGAA |  |  |
|  |  | F5’-GCTGTTGGACATCTACCGCAA | 1000bp |  |
|  |  | R5’-GAGGGCACGGTTGGCG |  | 60 |
|  |  | F5’-GTCTGAGGTGGACTACATGGAC | 1001bp | 60 |
|  |  | R5’- GGAACGGCATGTCCTCAA |  |  |
|  |  | F5’-GAAACGCAAGATCTCCGACG | 971bp | 60 |
|  |  | R5’ -TTGACGTCGAGCACGTAACT |  |  |
| **Ethambutol** | ***embB*** | F 5'-TGATATTCGGCTTCCTGCTC | 380bp | 58 |
|  |  | R 5’-ACCGCTCGATCAGCACATAG |  |  |
| **Quinolones** | ***gyrA*** | F 5'-GATGACAGACACGACGTTGC | 380bp | 58 |
|  |  | R 5'-GGGCTTCGGTGTACCTCAT |  |  |
|  | ***gyrB*** | F 5’-CCACCGACATCGGTGGATT | 420bp | 60 |
|  |  | R 5'-CTGCCACTTGAGTTTGTACA |  |  |
| **Injectables** | ***rrs-KAN^b^*** | F 5’- TATTAGACTGGCAGGGTCGC | 1536bp | 60 |
|  |  | R 5'- AAGTCCGAGTGTTGCCTCAG |  |  |
|  | ***eis*** | F 5'- TAGCACGGCCTTCAGAACTC | 1284bp | 60 |
|  |  | R 5'- GCCAGACACTGTCGTCGTAA |  |  |
|  | ***rpsL*** | F 5' CATGGCCGACAAACAGAACG | 479bp | 60 |
|  |  | R 5' CCGTAGACCGGGTCGTTG |  |  |
|  | ***gidB*** | F 5'CGATAGTTGAAGCCTGGCCC | 811bp | 61 |
|  |  | R 5'CGTCTCGAGAGCGGAGAATG |  |  |

PCR-polymerase chain reaction; Tm-annealing temperature

^a^F, forward primer; R, reverse primer. ^b^ *rrs-KAN* represented the amplified region containing the other hot spot conferring Kanamycin resistant in *rrs* gene.

**Table S3: PCR primer sequences used in MIRU-25/VNTR**

| **Locus** | **PCR primer pairs (5'-3')** | **PCR(H37Rv) product (bp)/copy numbers** | **Unit(bp)** |
| --- | --- | --- | --- |
| **MIRU2** | L)TGG ACT TGC AGC AAT GGA CCA ACT  R)TAC TCG GAC GCC GGC TCA AAA T | 508(2) | 53 |
| **MIRU4** | L)GCG CGA GAG CCC GAA CTG C  R)GCG CAG CAG AAA CGT CAG C | 352(3) | 77 |
| **MIRU10** | L)GTT CTT GAC CAA CTG CAG TCG TCC  R)GCC ACC TTG GTG ATC AGC TAC CT | 643(3) | 53 |
| **MIRU16** | L)TCG GTG ATC GGG TCC AGT CCA AGT A  R)CCC GTC GTG CAG CCC TGG TAC | 671(2) | 53 |
| **MIRU20** | L)GCC CTT CGA GTT AGT ATC GTC GGT T  R)CAA TCA CCG TTA CAT CGA CGT CAT C | 375(2) | 77 |
| **MIRU23** | L)CTG TCG ATG GCC GCA ACA AAA CG  R)AGC TCA ACG GGT TCG CCC TTT TGT C | 465(6) | 53 |
| **MIRU24** | L)CGA CCA AGA TGT GCA GGA ATA CAT  R)GGG CGA GTT GAG CTC ACA GAA | 447(1) | 54 |
| **MIRU26** | L)TAG GTC TAC CGT CGA AAT CTG TGA C  R)CAT AGG CGA CCA GGC GAA TAG | 438(3) | 51 |
| **MIRU27** | L)TCG AAA GCC TCT GCG TGC CAG TAA  R)GCG ATG TGA GCG TGC CACTCA A | 657(3) | 53 |
| **MIRU31** | L)ACT GAT TGG CTT CAT ACGGCT TTA  R)GTG CCG ACG TGG TCT TGAT | 651(3) | 53 |
| **MIRU39** | L)CGC ATC GAC AAA CTG GAG CCA AAC  R)CGG AAA CGT CTA CGC CCC ACA CAT | 646(2) | 53 |
| **MIRU40** | L)GGG TTG CTG GAT GAC AAC GTG T  R)GGG TGA TCT CGG CGA AAT CAG ATA | 408(1) | 54 |
| **VNTR1955** | L)AGA CGT CAG ATC CCA GTT  R)ACC CGA CAA CAA GCC CA | 267(1) | 57 |
| **VNTR2347** | L)GCC AGC CGC CGT GCA TAA ACC T  R)AGC CAC CCG GTG TGC CTT GTA TGA C | 563(3) | 57 |
| **ETRC** | L)GTG AGT CGC TGC AGA ACC TGC AG  R)GGC GTC TTG ACC TCC ACG AGT G | 276(3) | 58 |
| **Mtub39** | L)CGG TGG AGG CGA TGA ACG TCT TC  R)TAG AGC GGC ACG GGG GAA AGC TTA G | 388(2) | 58 |
| **Mtub30** | L)CTT GAA GCC CCG GTC TCA TCT GT  R)ACT TGA ACC CCC ACG CCC ATT AGT A | 363(1) | 58 |
| **VNTR4120** | L)GTT CAC CGG AGC CAA CC  R)GAG GTG GTT TCG TGG TCG | 447(2) | 57 |
| **VNTR3820** | L)TGC GCG GTG AAT GAG ACG  R)ACC TTC ATC CTT GGC GAC | 444(3) | 57 |
| **Mtub04** | L)CTT GGC CGG CAT CAA GCG CAT TAT T  R)GGC AGC AGA GCC CGG GAT TCT TC | 639(2) | 51 |
| **ETRB** | L)ATG GCC ACC CGA TAC CGC TTC AGT  R)CGA CGG GCC ATC TTG GAT CAG CTA C | 518(3) | 57 |
| **Qub26** | L)GTG CCG GCC AGG TCC TTC C  R)CAC CGC GTG TTT GAC CCG AAC | 731(5) | 111 |
| **ETRA** | L)ATT TCG ATC GGG ATG TTG AT  R)TCG GTC CCA TCA CCT TCT TA | 397(3) | 75 |
| **Mtub-34** | L)GGT GCG CAC CTG CTC CAG ATA A  R)GGC TCT CAT TGC TGG AGG GTT GTA C | 488(2) | 54 |
| **Qub11b** | L)CCG ATG TAG CCC GTG AAG A  R)AGG GTC TGA TTG GCT ACT CA | 547(5) | 69 |

PCR – polymerase chain reaction
